# Urine with stones: nephrocalcinosis and vitamin D

**DOI:** 10.1186/1824-7288-40-S1-A18

**Published:** 2014-08-11

**Authors:** Luisa Santangelo, Mario Giordano

**Affiliations:** 1 Department of Pediatrics, Unit of Paediatric Nephrology, University Hospital “Policlinico Consorziale - Giovanni XXIII”, Bari, Italy

## Background

Nephrocalcinosis (NC) refers to diffuse, fine, renal parenchymal calcification, as assessed by radiology or ultrasonography. In medullary NC, the calcification tends to occur in the area of the renal pyramids. It is typically associated with elevated urinary calcium, phosphate, and oxalate, or it can occur with alkaline urine. Any disorder that can lead to hypercalcemia or hypercalciuria may be implicated. Instead of stone formation, smaller parenchymal calcifications are deposited in the medulla, usually bilateral and symmetric.

Vitamin D hormones play a central role in calcium homeostasis. Tight control of the vitamin D system requires inactivation of its active compound 1,25-dihydroxyvitamin D3 (1,25(OH)2D3) through 24-hydroxylation by means of the enzyme 24-hydroxylase (CYP24A1) and degradation to calcitroic acid [1].

Recently loss-of-function mutations of vitamin D-24 hydroxylase (gene CYP24A1) have been recognized as a cause of hypercalcaemia and NC/nephrolithiasis in infants and adults suffering from Idiopathic Infantile Hypercalcaemia (IIH) [2]. Prevalence and natural history of this condition are still to be defined also in pediatric patients. We investigated the prevalence of CYP24A1 mutations in a cohort of pediatric patients with NC referred to our outpatient service from January 2012 to December 2013.

## Materials and methods

Nineteen children with NC were investigated, by measuring calcium, phosphate, and vitamin D, and by performing CYP24A1 mutation analysis.

## Results

All patients were diagnosed with NC after ultrasonographic assessment at a mean age of 3,5 years and were analyzed with the diagnostic flowchart proposed by Ammenti [3] for detecting causes of NC. Sequence analysis of CYP24A1, which encodes 25-hydroxyvitamin D 24-hydroxylase, the key enzyme of 1,25-dihydroxyvitamin D(3) degradation, revealed mutations in three affected children, two were compound heterozygous for their specific mutations and one presented only one mutation in a single allele.

All the affected patients showed hypercalciuria, while only compound heterozygous patients showed chronic hypercalcaemia with depressed parathyroid hormone (PTH).

All the patients had normal renal function, despite high-grade NC, and were treated with potassium citrate to restore normal citraturia. Of note all patients suffered of recurrent urinary tract infections, which disappeared after normalization of calciuria due to potassium citrate supplementation.

## Conclusion

The results of this study suggest a close relationship between CYP24A1 mutations, and onset of NC in pediatric patients. However further studies are needed to determine the prevalence of CYP24A1 mutation in NC patients, to define natural history and to develop novel therapeutic approaches.
